# Into Your (S)Kin: Toward a Comprehensive Conception of Empathy

**DOI:** 10.3389/fpsyg.2020.531688

**Published:** 2021-01-12

**Authors:** Tue Emil Öhler Søvsø, Kirstin Burckhardt

**Affiliations:** ^1^Department of Philosophy and Humanities, Institute of Greek and Latin Languages and Literatures, Freie Universität Berlin, Berlin, Germany; ^2^Department of Philosophy, Humboldt Universität zu Berlin, Berlin, Germany; ^3^Clinic for Psychosomatic Medicine and Psychotherapy, Städtisches Klinikum Görlitz, Görlitz, Germany

**Keywords:** empathy, stoicism, embodied cognition, attachment, phenomenology, prosocial motivation, affective intentionality

## Abstract

This paper argues for a comprehensive conception of empathy as comprising epistemic, affective, and motivational elements and introduces the ancient Stoic theory of attachment (Greek, *oikeiōsis*) as a model for describing the embodied, emotional response to others that we take to be distinctive of empathy. Our argument entails that in order to provide a suitable conceptual framework for the interdisciplinary study of empathy one must extend the scope of recent “simulationalist” and “enactivist” accounts of empathy in two important respects. First, against the enactivist assumption that human mindreading capacities primarily rely on an immediate, quasi-perceptual understanding of other’s intentional states, we draw on Alfred Schutz’ analysis of social understanding to argue that reflective types of understanding play a distinct, but equally fundamental role in empathic engagements. Second, we insist that empathy also involves an affective response toward the other and their situation (as the empathizer perceives this). We suggest analyzing this response in terms of the Stoic concepts of attachment, concern, and a fundamental type of prosocial motivation, that can best be described as an “extended partiality.” By way of conclusion, we integrate the above concepts into a comprehensive conceptual framework for the study of empathy and briefly relate them to current debates about empathic perception and prosocial motivation. The result, we argue, is an account that stays neutral with regard to the exact nature of the processes involved in producing empathy and can therefore accommodate discussion across theoretical divides—e.g., those between enactivist, simulationalist, and so-called theory-theorist approaches.

## Introduction

“Slipping into someone else’s skin”—this bodily metaphor serves as an almost dictionary definition of empathy. In general, the body looms large when speaking about empathy and kindness. We “put ourselves in someone else’s shoes” or “listen to our hearts” and the excessively empathic among us have “a bleeding heart.” Nonetheless, the body occupies a somewhat un-easy place in modern discussions of empathy, which have tended to focus on just one part of the bodies involved, namely the brain of the empathizer.

With inspiration from phenomenology and interaction theory, recent accounts of empathy have pushed to transcend this focus on a more or less isolated mind, emphasizing the immediate, and “intercorporeal” nature of empathic interaction (see e.g., Fuchs and De Jaegher, 2009; Zahavi, 2011, 2014; Gallagher, 2012a; Ciaunica, 2017; Fuchs, 2017; for a good review, see Zahavi and Michael, 2018). These phenomenological and “enactivist” accounts construe empathy as a basic type of other-directed intentionality that enables us to directly perceive the mental states of others through our interaction with them. They thus form part of a broader research agenda known as 4E cognition committed to studying our cognitive processes as embodied, extended, enactive, and embedded phenomena (for a concise introduction, see Newen et al., 2018).

This conception of the mind as encompassing and in part co-constituted by the body and its environment carries great prospects for the study of interpersonal processes but arguably 4E analyses of empathy remain underdeveloped in crucial respects. While these phenomenologically inspired discussions of empathy have broadened the scope on empathy by conceiving of it as an embodied and enactive phenomenon, they have also tended to focus exclusively on its epistemic aspects neglecting the affective aspects at the very root of the word and broadly considered to be central to empathy. The aim of the present paper is to establish a conceptual framework that allows for a more comprehensive analysis of empathy as both an epistemic and an emotional phenomenon.

More specifically we propose to supplement the enactivist account of basic empathy with an analysis of the affective attitude and response involved in empathic engagements. We develop this analysis with inspiration from the ancient Stoics’ account of affective intentionality, known as their theory of *oikeiōsis*. On this theory, the bodily processes, pointed to in enactivist accounts of empathy as enabling an immediate other-understanding, are instead seen as producing an attitude of attachment, concern, and extended partiality which provides the affective foundations of interpersonal relations. The Stoic analysis of embodied affectivity therefore nicely complements the traditional phenomenological analysis of empathy’s epistemic aspects and provides the conceptual means for relating the enactivist account to the ongoing debates about empathy’s role in motivating prosocial behavior.

In the section “Empathy: Cognitive, Emotional, Primary, and Extended” by briefly situating the enactivist approach within the broader debates about empathy and social understanding and introduce one of the most extensive accounts of enactive empathy, namely the one developed by the German philosopher and psychiatrist Thomas Fuchs. In the section “Social Understanding: Enactive and Reflective” we discuss the concept of bodily resonance, taken by Fuchs to underlie our empathic capacities, in more detail drawing on the early phenomenologist Alfred Schutz’s (1967) analysis of social understanding and meaning to point out the limits of such “enactive” understanding and contrast it with explicit, “reflective” types of social understanding. In the subsequent section, we then introduce the Stoic theory of *oikeiōsis* and interpersonal relations and compare this account of affective intentionality with the one underlying Fuchs’ account of empathy. Having thus delineated the epistemic and affective scope of empathy, in the section “Discussion: Toward a Comprehensive Conception of Empathy” we piece together our account of empathy specifying the individual processes we take it to involve and briefly relating it to existing, empirical debates about the attachment toward one’s own body (so-called “Body Ownership”) and the nature of the prosocial motivation produced by empathy.

## Empathy: Cognitive, Emotional, Primary, and Extended

Before zooming in on the many debates surrounding the concept of empathy, it may be useful to get a rough idea about what kind of thing(s) the term “empathy” is generally taken to refer to and how we shall use it. Batson (2009) has distinguished two questions that students of empathy have been interested in: “How can one know what another person is thinking and feeling?” and “What leads one person to respond with sensitivity and care to the suffering of another?” (Batson, 2009, p. 3). He identifies eight different psychological states that have all been called empathy and seen as relevant to answering one or both of the questions above. These states are often subsumed under the terms emotional and cognitive empathy, i.e., the ability to resonate with the emotions of others or “feel with them” and the appreciation of their emotions through purely cognitive means, also called perspective taking. Batson sees this complexity as a basic fact about the study of empathy that one needs to acknowledge and deal with as best one can.

Our strategy for dealing with this complexity is to distinguish between empathy as a complex emotional phenomenon and the various empathic processes that may go into producing it. This means that we will take a top-down approach to empathy analyzing it in its developed form as it appears relatively late in childhood and largely stay at the purely conceptual level of determining what epistemic, emotional, and motivational states empathy involves.

We tentatively define empathy as *a benevolent engagement with the affective states of others which provides us with a grasp of their state and produces an affective response within our body*. This engagement, we take it, plausibly involves a whole range of distinct, empathic processes, and states, which complement, interrelate, and inform each other in intricate ways, which we are really only beginning to grasp and describe scientifically (Batson, 2009; Zaki and Ochsner, 2012). The purpose of viewing these various processes as elements of empathy and not in isolation is to allow us to study their interaction and interrelations, not just their individual workings, on the assumption that there might be more to the whole than the sum of its parts (cf. Zaki and Ochsner, 2012).

Our definition of empathy is deliberately vague, and in the first instance, it only aims to provide a possibly neutral language for speaking about the individual aspects of empathy while allowing us to distinguish it from distinct, but closely related phenomena such as emotional contagion, sharing, and mindreading^1^. To delineate the scope of our definition it may be useful to compare it to some recent, prominent attempts to define empathy.

Frédérique de Vignemont, along with shifting co-authors, has developed a highly systematic account of empathy and proposed a set of necessary and sufficient conditions that distinguish empathy from related phenomena (de Vignemont and Jacob, 2012; cf. de Vignemont and Singer, 2006). These are:

1. the affectivity condition: both the empathizer and the target of her empathy experiences an affective state
2. the interpersonal similarity condition: empathizer and target person must experience similar affective states
3. the causal path condition: the affective state of the empathizer must be caused by the affective state of the target person
4. the ascription condition: the empathizer must be aware of the target person’s affective state and of the fact that this state is the cause of her own affective state
5. the caring condition: the empathizer must care about the target person’s affective life

Our definition retains condition (i), (iii), and (v) by insisting that for an engagement to qualify as empathic it must be concerned with the affective state of someone else, it must produce an “affective response” in the empathizer, and it must be “benevolent^2^.” Similar to de Vignemont, we thus distinguish empathy from standard mindreading (in the sense of merely registering the intentional states of others) and what is sometimes simply called sharing [where two people experience the same affective state but caused by the same intentional object (Scheler, 1954)].

Condition (iv) we only accept in a slightly weaker form. By speaking of a grasp rather than an explicit awareness of the other’s state we thus wish to include the pre-reflective sensing and reacting to others’ states, sometimes called “basic empathy” (Stueber, 2006; Fernandez and Zahavi, 2020), as instances of empathy along with the conscious ascriptions exclusively recognized by de Vignemont. This pre-reflective awareness of the other’s state is still sufficient to distinguish empathy from emotional contagion, i.e., the unconscious adoption of others’ affective states as witnessed e.g., in the spreading of mass panic (Scheler, 1954), which does not imply any awareness of others’ states.

Condition (ii), the interpersonal similarity condition, is far more controversial and in effect limits the scope of empathy to cases of vicarious sharing. De Vignemont describes her account as “simulation-based” and commits herself to the view that empathy relies on the recognition of mental states through an internal simulation or enactment-imagination of that state, which is then ascribed to the other (de Vignemont and Jacob, 2012). This narrowly simulationalist conception of empathy is problematic since it arguably misconstrues the scope of empathic understanding. As Dan Zahavi argues: “To insist that the empathizer must have the same (kind of) state as the target, is to miss what is distinctive about empathy, namely the fact that it confronts you with the presence of an experience that you are not living through yourself” (Zahavi and Michael, 2018, p. 597).

By contrast, Zahavi has suggested a definition of empathy as “a distinctive form of other-directed intentionality, distinct from both self-awareness and ordinary object-intentionality, which allows foreign experiences to disclose themselves as foreign rather than as own” (Zahavi, 2014, p. 138). This definition of empathy as a distinct type of intentionality, in turn, restricts the scope empathy to its epistemic aspects (i.e., Batson’s first question) and allows for empathy to appear in combination with any number of affective states and attitudes toward others. Indeed, Zahavi explicitly bites the bullet that on such an account “it is perfectly coherent to think that an expert torturer may rely on empathy in order to work out how best to push her victim’s buttons” (Zahavi and Michael, 2018, p. 597).

This narrowly epistemic conception of empathy has a long tradition within philosophy—especially phenomenological philosophy where the concept originally evolved (Stueber, 2006; Zahavi, 2010)—and it creates a neat distinction between empathy and such clearly affective phenomena as sympathy or compassion. But it also blurs the line between empathy and mindreading more broadly and goes against the common intuition that empathy somehow implies a basic pro-attitude toward the other. Since this intuition is strong enough to have inspired an entire strand of research on empathy (i.e., the studies of Batson’s second question), we believe that it should be reflected in the definition of the concept.

In contrast to Zahavi and following de Vignemont, we thus agree that empathy is a basically benevolent way of engaging with others where both empathizer and target person experience an affective state. Unlike de Vignemont, however, we do not assume that these states must be similar or that the state of the empathizer has to serve as her basis for understanding the target’s state. It is sufficient that it is a response elicited by the engagement with that state (through simulation or otherwise).

In defining empathy as an engagement which both reveals the target’s affective state *and* produces an affective response in the empathizer, we thus distinguish empathy from mindreading more broadly both in terms of its proper object and its effect. Empathy, on our view, is a type of mindreading that provides understanding of the affective states of others (widely construed as any state that involves the ascription of affective value, cf. Fuchs’ concept of emotions discussed below, section “Social Understanding: Enactive, and Reflective”), whereas mindreading as such can deal with all types of mental states, including non-valenced beliefs. Also, empathy requires you to feel something for the person you empathize with, not just neutrally (or maliciously) registering their affective state (cf. Gallagher, 2012a who talks of empathy as involving a “primary and irreducible affective state—the state of feeling empathy.”). Empathy, on our account, not only involves understanding, but also an affective response to the state of the target person.

It should be clear by now that we cast the net wider than many other students of empathy. As the preceding remarks convey, we see no reason to restrict the concept to a purely epistemic other-comprehension or associating it narrowly with a particular type of other-comprehension. On our account, the phenomena described by Zahavi and de Vignemont are thus possible elements of empathy, but what sets empathy apart is the combination of its epistemic and affective elements. In what follows we shall try to develop an account that encompasses all these elements. Since the majority of work on empathy has focused on questions about the possibility of other-comprehension (Batson, 2009; Zaki and Ochsner, 2012), we shall start with a discussion of the epistemic aspects of empathy before we move on, in the section “Affective Intentionality: Bodily Resonance and Stoic Attachment” and “Discussion: Toward a Comprehensive Conception of Empathy,” to discuss the affective responses involved in empathy and how these interrelate with its epistemic aspects.

Within discussions of social understanding “empathy” is often used more or less synonymously with the term “mindreading” as an answer to Batson’s first question of how we can understand others^3^. This has traditionally been explained in terms of “theory of mind” (ToM), i.e., the basic insight that other people have minds and the abilities to reconstruct the inner life of others that follows upon this insight. It is controversial, however, whether these abilities are best understood as a type of inference, the so-called theory-theory, or as a type of simulation, the so-called simulation-theory (for good surveys of the debate, see Stueber, 2006, chap. 3; Gallagher, 2012b). Against this a growing number of researchers working within broadly phenomenological traditions have objected that these explanatory models unnecessarily complicate the matter by ignoring the extent to which the “inner” states of others are directly perceptible to us (Jensen and Moran, 2012; Gangopadhyay, 2014 provide good overviews of this critique).

This debate is sometimes described in terms of the different perspectives the individual approaches favor. Simulation-theory thus assigns the first-person perspective a fundamental significance in enabling social understanding. On this view, it is the ability to imagine yourself being in a certain state and then project this state onto others which explains the human ability to recognize what others are thinking and feeling (see e.g., Goldman, 2006; Stueber, 2006; de Vignemont and Jacob, 2012; Gallese and Sinigaglia, 2018). Theory-theory, on the other hand, likens the interpretation of others’ behavior and inner states to the application of a scientific theory. On this view, social understanding relies on inferences about other people’s behavior, facial expressions etc. based on a theoretical knowledge about how people normally think and feel when they behave in certain ways. This puts the empathizer in the role of an observer viewing the target person in a third-person perspective, drawing more or less objective inferences based on how s/he behaves (see e.g., Premack and Woodruff, 1978; Gopnik and Wellman, 1992; Carruthers and Smith, 1996). Finally, phenomenological and enactivist approaches to social understanding have stressed the fact that we normally engage with the thoughts and feelings of others in the context of direct social interaction where we experience the other in an immediate and interactive second-person perspective. Through the sensorimotor coordination and mutual attunement that characterizes direct, physical interaction the actions and intentions of others are immediately intelligible to us and this enables a direct or “primary” type of intersubjective understanding that does not rely on any inferences or oblique ascriptions regarding their mental states (see e.g., Gallagher, 2004, 2012b; Hutto, 2004; Zahavi, 2008; Fuchs and De Jaegher, 2009).

The basic assumption underlying the enactivist approach is therefore that “the mind of the other is not entirely hidden or private, but is given and manifest in the other person’s embodied comportment,” (Gallagher, 2004, 204; cf. Fuchs and De Jaegher, 2009, p. 469). As Gallagher points out it is only when “everyday second-person interactions break down, or when I have problems understanding the other person, I may engage in a specialized theoretical approach that appeals to third-person explanation or prediction. But such specialized cognitive approaches do not characterize our primary or everyday encounters with others” (Fuchs and De Jaegher, 2009, p. 202). Instead of relying only on traditional “cognitivist” approaches, social understanding therefore needs to be studied in terms of the enactive and embodied processes it involves (Fuchs and De Jaegher, 2009; Froese and Fuchs, 2012; Gallagher, 2012b; De Jaegher et al., 2017).

This enactive approach to social understanding has gained considerable traction within recent years and, we believe, it also offers the most promising starting point for an investigation of empathy. The most comprehensive, enactivist account of empathy to date is arguably the series of articles published individually and in collaboration with other leading researchers within the field by Thomas Fuchs, who draws extensively on both enactive and phenomenological discussions of social understanding (see most importantly Fuchs and De Jaegher, 2009; Froese and Fuchs, 2012; Fuchs and Koch, 2014; Fuchs, 2016, 2017).

Fuchs, like Zahavi, conceives of empathy as an immediate perception of the other’s intentional state. He describes this as relying on “mutual incorporation” (Fuchs and De Jaegher, 2009; Fuchs, 2016) which constitutes a form of interaffectivity or “interbodily resonance,” i.e., a mutual attunement or coordination in both movements and affections between interacting subjects (Fuchs and De Jaegher, 2009, p. 472–4; Fuchs and Koch, 2014, p. 5–7). Importantly, Fuchs here assumes that the immediate physical reaction to the other’s expressions, e.g., the mirroring of a smile or the jolt away from an angry roar, is co-constitutive of the affective state we experience, in this case joy or fear. A feedback-cycle of affective responses can therefore arise: each participant picks up and reacts to the state of the other who then in turn reacts to her state (Fuchs and Koch, 2014).

The reciprocal and immediate nature of such interaction allows for participatory sense-making and an intimate attunement of emotional states. The interaction with others therefore provides an immediate grasp on their emotional states and intentions-in-action because we perceive and participate directly in them (Fuchs and De Jaegher, 2009; Fuchs, 2013; Fuchs and Koch, 2014). As Fuchs stresses, the sensorimotor coordination happens pre-reflectively and more or less instantaneously and the dynamics of this inter-affective system becomes “highly autonomous and are not directly controlled by the partners” (Froese and Fuchs, 2012, 213; see also Fuchs and De Jaegher, 2009; Fuchs, 2016). Fuchs therefore concludes: “Thus, emotions are not inner states that we experience only individually or that we have to decode in others, but primarily *shared states* that we experience through interbodily affection” (Fuchs and Koch, 2014, p. 7, their emphasis).

In contrast to de Vignemont’s simulation-based account of empathy, Fuchs does not assume that the state of the empathizer and target person have to be the same. They are shared in the sense that they have their common origin in the dynamic interaction, but this can also involve complementary reactions. Also, we do not have to become aware of the shared state or explicitly ascribe it to the other in order to empathize. The understanding characteristic of this intercorporeality can of course be made explicit and reflective, but Fuchs emphasizes its fundamentally pre-reflective, implicit nature (Fuchs and De Jaegher, 2009; Froese and Fuchs, 2012; Fuchs, 2017). Supplemented by what Fuchs calls bodily memory, i.e., an implicit, pre-reflective memory which encapsulates both the interactive patterns of particular relationships and a more general knowledge of how to interact with others, this enables a fundamental kind of intersubjective understanding which Fuchs identifies as “primary empathy” (Fuchs, 2016, 2017).

Fuchs seems to assume that this basic form of empathy scales straight forwardly into more explicit and reflective attempts to understand others, like the ones subsumed under de Vignemont’s definition of empathy, without the need for any further processes. He thus contrasts primary empathy with any attempt to reconstruct the inner states of others by means of logical inferences or simulation, which he calls “extended empathy” (Fuchs, 2013, 2017). According to Fuchs, these cognitive mechanisms are mainly relevant in cases of ambiguity or misunderstanding, whereas primary empathy sufficiently accounts for our everyday social interactions (Fuchs, 2017). Extended empathy appears to be a last resort or “deprived” version of primary empathy (Fuchs and De Jaegher, 2009, p. 472).

Fuchs’ account of primary empathy thus ascribes both psychological and epistemic immediacy to the grasp of others’ affective states provided through direct perception, i.e., it neither involves further psychological mechanisms (such as inference, projection or explicit ascription) nor does it require any further epistemic justification (as provided by e.g., ToM and “folk psychology”). His demotion of the cognitive mechanisms associated with extended empathy to a purely auxiliary or supplementary function, moreover, suggests that he takes this immediacy to characterize the majority of the social understanding involved in everyday interaction. This is a strong claim and, as we argue in the following section, it must be strongly modified.

While we take Fuchs’ account of empathy with its basis in interbodily resonance and mutual incorporation to offer a promising way of capturing the embodied and enactive character of empathic engagements, the scope of the understanding provided by such engagements is in our view more limited than he appears to acknowledge. Based on a critical discussion of the forms and limits of social understanding informed by Schutz’s phenomenological analysis of intersubjective meaning, the following section argues that even in everyday interaction empathic understanding involves a complex interplay between various processes, including both primary empathy and ToM. The fundamental significance of interbodily resonance and mutual incorporation for empathy, however, is not restricted to the epistemic aspects highlighted by Fuchs, but is equally, or perhaps even more closely, tied to their role in shaping our affective response to others.

## Social Understanding: Enactive and Reflective

Before we move on let us look more carefully at the account of embodied intentionality underlying Fuchs’ account of primary empathy. On this account, empathy relies on our body’s general capacity to resonate with its environment. This is brought out in his discussion of bodily resonance and embodied affectivity (Fuchs and Koch, 2014). As Fuchs stresses “bodily feelings should never be conceived as a mere by-product or add-on, distinct from the emotion as such, but as the *very medium* of affective intentionality.” (Fuchs and Koch, 2014, p. 3 *sic*). By positing a distinctively bodily format for our affective intentionality Fuchs assigns the body a fundamental role in shaping how we perceive the world, allowing him to distinguish between a primary, embodied affectivity and the reflective processes that rely on it (cf. Goldman and de Vignemont, 2009 on this distinction between embodied and reflective processes).

The nature of bodily feelings, however, is twofold. Every feeling is simultaneously an affection and an expression. It registers an affective quality of the environment through e.g., visual, olfactory or tactile input, while the bodily changes this involves (facial, gestural, tensional etc.) also express our reaction to these “affective affordances” often including a tendency to move in certain ways, e.g., toward or away from an object (a tendency Fuchs dubs an “e-motion”). It is this registration-cum-reaction that Fuchs refers to by the broad term “emotion.” Emotions, according to Fuchs, are thus the key to understanding intentional action: “Without emotions, the world would be without meaning or significance; nothing would attract or repel us and motivate us to act” (Fuchs and Koch, 2014, p. 2).

When we interact with other living subjects our intrabodily resonance becomes entwined in a reciprocal process of affection and expression: I pick up on your expressions and inevitably react to them, if even in the slightest of ways, and my reaction in turn impacts you and elicits a new expression on your part. Like our intra-bodily resonance, this inter-bodily resonance is circular, but it extends beyond the individual to include all the interacting subjects thereby creating lesser or stronger feelings of “mutual incorporation” among them. This intercorporeality and sharing of states, as we saw above, are what enable empathic understanding on Fuchs account (Fuchs and Koch, 2014, p. 5–7).

As Fuchs and colleagues convincingly argue, the sensorimotor coordination and interbodily resonance involved in direct interaction thus provides an immediate access to the emotions and intentions of others in the sense of making these directly perceptible to us (for a review of recent studies into the neuronal bases of these mechanisms offering an interpretation in more simulationalist terms, see Gallese and Sinigaglia, 2018; cf. Stueber, 2006, p. 131–152). When we interact with others the majority of the movements, facial expressions, odors etc. they exhibit do not seem to require the inference of a hidden inner state in order for them to acquire meaning. In an important sense, your expression really is your emotion (recall the definition of bodily feelings as both affections and expressions). Thanks to the human ability to register and react to the bodily feelings of other living subjects their expressions therefore appear meaningful in the same immediate way that a piece of charcoal appears black and a stone hard.

This conception of interbodily resonance is capable of accounting for much of the interpersonal understanding involved in everyday encounters. It thus seems plausible that e.g., the yelling and tenseness of another person is directly perceived as “anger” rather than as indications allowing us to infer that the person in question is angry. But as we suggested above, there are also important limitations to such understanding. The basic contours of these limitations were brought out by Alfred Schutz in his penetrating analysis of social understanding (1967, esp. 20–38).

Schutz’s point of departure is a critique of Scheler (1954) who, much like Fuchs, had pointed to the immediate, pre-reflective insight into the states of others offered by bodily expressions as a basis for empathy. Schutz, however, insists that this understanding is more limited than Scheler acknowledges (on this early phenomenological debate about empathy and Schutz’ contribution to it, see Zahavi, 2010). First of all, not all expressions are also an attempt to express something, and even when this is the case, the agent’s expressions only offer an indirect insight into their intentions: seeing someone who is yelling and raging, we immediately understand that the person is angry, but understanding their anger is more complicated. Grasping the full meaning of this state involves placing the agent’s expressions within a broader context of meaning and interpreting them in that light. This is where things get murky (Schutz, 1967, p. 22–24).

To elucidate this interpretive process Schutz, building on Husserl’s distinction between “that which is meant” (*Bedeutung*) and the act of “meaning” (*Bedeuten*), distinguishes between a level of “objective” meaning and one of “subjective” or “intended meaning” (*intendierter Sinn*) (Schutz, 1967, 33ff.). As he emphasizes the interpretive schemata we apply to the expressions of others (as well as our own) are to a large extent shared, intersubjective and in that sense “objective,” but this intersubjectivity always presupposes an individual act of meaning-endowment (*Sinngebung*) (see further Schutz, 1967, chap. 2). You and I may therefore agree about the meaning of a given expression but we do so on the basis of distinct, and possibly differing, acts of meaning. My subjective experience will therefore necessarily differ from yours simply by virtue of it being mine and not yours.

A basic upshot of this distinction, as we interpret it, is that there are two levels on which you may strive to understand the meaning others experience: understanding it at the properly intersubjective level of identifying the meaning-content of their expressions, actions and intentions; and understanding how they experience this content, which remains essentially inaccessible, because this relies on an intentional operation of their consciousness that most often eludes even the awareness of the agent herself (Schutz, 1967, p. 34–37). The point of this distinction is not that social understanding is impossible, but simply that one should be aware that the meaning one is ascribing to others is of the intersubjective kind and as such a product of one’s own intentionality—not the subjective meaning of the person one is trying to understand (Schutz, 1967, p. 30–31 and esp., 37–38).

We shall not go into any detail about the elaborate and sophisticated account of social understanding Schutz goes on to develop, but only offer a few selective observations (see Schutz, 1967, chap. 3; with Zahavi, 2010; León and Zahavi, 2016). For Schutz, the lived experience prior to any act of meaning-endowment is the privileged level of experience and we can crucially share this immediate experience of our existence with others through direct interaction, interlocking our intentionality with theirs and sensing their stream of consciousness. This immediate and pre-reflective other-comprehension (*Fremdverstehen*) is the closest one gets to the subjective meaning of others. The very act of meaning-endowment, by contrast, is reflective in the very basic sense that it imposes a certain meaning on our immediate perception. The moment we begin assigning meaning to the experience of others, we are therefore engaging in a private act of reflection rather than the special type of perception associated with true other-comprehension.

Corresponding to the sharing and interlocking of intentionality, which Schutz regarded as characteristic of true other-comprehension, Fuchs speaks of mutual incorporation as involving a decentering of our intentionality (Fuchs and De Jaegher, 2009, p. 476). Like Schutz, he assumes that this direct engagement offers an immediate and often quite intimate experience of the other’s consciousness. Unlike Schutz, however, he seems to assume that it also grants a quite extensive, immediate understanding of the meaning they assign to their experience. This, Schutz would insist, is where we must distinguish between subjective and intersubjective meaning.

On a Schutzian analysis, what we share in cases of mutual incorporation is strictly speaking not our intentionality, in the sense of directly participating in each other’s acts of meaning-endowment. Instead we attune and align our individual meaning-endowments and then share the meaning-content constituted by these intentional operations. Thanks to the intersubjective character of the meaning-content each of us assign to our experiences, we are likely to assign similar content to many of the experiences we share during our interaction, thereby making your acts of meaning-endowment (including your emotions in Fuchs’ broad sense) immediately intelligible to me. But this is due to a contingent overlap in the individual acts of meaning-endowment that each of us perform and the greater the difference in our past experience, cultural background etc., the greater the risk that our acts of meaning-endowment drift apart and I end up misunderstanding you.

Such misunderstandings are often quite subtle and of little practical importance to our social interaction and as such they pose no threat to Fuchs’ claim that the immediate understanding derived from direct perception provides the predominant basis for everyday human interaction. The very negligibility of our misunderstandings, however, also points to a fundamental limitation in Fuchs’ account of empathy: how can we know whether we really understand the other’s experience or not? Since direct perception offers no access to the process of meaning-endowment, it provides no independent justification for our ascriptions of meaning-content to the experience of others and no means for detecting possible errors—except for the one’s that incidentally disclose themselves in the course of our interaction. We are therefore liable to miss the often quite subtle differences between our own perspective and that of the other.

The “interbodily resonance” and “bodily memory” that Fuchs points to as enabling our direct perception of others’ intentional states certainly provides some justification for the empathizer’s assignment of meaning-content to the target’s state (cf. Stueber, 2006, p. 142–147). Even though the human capacities to interact with and understand others in immediate and pre-reflective ways are indeed impressive, however, they can only take us so far. Consider a 3 year-old giving her father a picture she has painted. It is immensely plausible that due to their interactive history she possesses an “implicit,” bodily knowledge of his reaction (Fuchs and De Jaegher, 2009; Fuchs, 2016; cf. Lyons-Ruth et al., 1998), that creates an immediate understanding and maybe even an anticipation of his reaction—her entire bodily posture reflects her enjoyment of the gratitude and compliments she is about to receive, even before the picture has left her hands. But all this—and this really is quite a bit of complex, social understanding—tells the child nothing about whether her father’s reaction is caused by the picture, her act of generosity, or whether he is just being polite in order not to let her expectations down.

Fuchs never points to any mechanisms capable of providing such understanding. Empathy as he construes it may therefore provide us with a fairly reliable insight into what others experience, but arguably, his exclusive focus on recognitional capacities fails to acknowledge that we are not always just after the “what” of others’ intentional states. Quite often the question driving our interest is how they came to see things or act this way. Moreover, these two types of understanding are plausibly seen as mutually informing: if my daughter gets the suspicion that I am dissimulating my joy, this conscious and reflective suspicion is likely to impact her immediate bodily reaction to my expressions of joy, regardless of the sincerity of my expressions (cf. Schmidsberger and Löffler-Stastka, 2018 who raise a similar point against Fuchs).

As opposed to the parallel assignment of the same content to a shared experience, we therefore suggest, the attempt to grasp the meaning-content of others, as this really appears to them, is a more ambitious task to the degree that it aims at understanding not just what the other intends, but also how and why they intend it. This involves a broadening in the scope of our other-directed intentionality from a sole focus on the meaning-content of the target’s state to include the act of meaning-endowment producing that content.

Given that meaning-endowments are not directly observable, mostly eluding even the subjects own introspective “glance,” reflecting on them is likely to include more than the direct perception of their effects. Such understanding, it would seem, relies on considerations about the context and processes informing the target’s act of meaning-endowment as entailed by ToM and “folk psychology” (Stueber, 2006, chap. 4) or narrative competence (Gallagher, 2012a). We should therefore not, as suggested by Fuchs, conceive of these reflective, cognitive mechanisms as mere extensions or “deprived” versions of our immediate perceptual capacities, but as granting their own distinctive type of social understanding.

The very operation of trying to understand the meaning-endowments of others thus bears a close affinity to the act by which we focus attention on our own acts of meaning-endowment. Understanding e.g., why I reacted with fury in a given situation is not that different from understanding why you did so. Both operations necessarily focus on already elapsed (or future) experiences and both imply a shift from the engaged and immediate experience of the world to a sort of meta-perspective on how we/the other experience it. Reflection thus imposes a certain distance and loss of immediacy, but in contrast to the heuristic methods of direct perception, it also opens our understanding of the other to conscious evaluation and revision and thereby enables a dialogue between the interacting subjects.

In this sense, the reflective engagement with the experiences of others may be said to offer a deeper and more truly interpersonal understanding. Whereas the first-person experience of your intentionality is strictly speaking only accessible to you (with the possibilities of interpersonal resonance and shared meaning production opened up by direct interaction), it thus seems possible that others possess a deeper reflective understanding of your acts of meaning-endowment than you do yourself. After all, it is not uncommon that it takes an outside observer to make you aware that you are attributing a certain meaning to certain phenomena. In that instance the outside observer, in virtue of her correct hypothesis about your unconsciously experienced meaning, can reasonable be said to have possessed a deeper understanding of your intentional state than you did yourself.

This suggests that both with regard to self- and other-awareness we should recognize two distinct ways of understanding an intentional state: an immediate, perceptual grasp and a reflective interpretation [cf. Stueber’s (2006) distinction between basic and reenactive empathy and Gallagher’s (Gallagher, 2012a,b) distinction between “low-level” and “high-level” processes]. One might, we suggest, speak of an “enactive” and a “reflective” understanding of others, thereby highlighting the immediate, interactive, and embedded character of the former, which derives from interbodily resonance, and the meta-perspective on the other’s intentional states implied by the latter.

Empathy, as an other-awareness directed specifically at the affective states of others, aims at both these types of understanding, we take it, but to variable degrees depending on the context. We have pointed to Fuchs’ analysis of interbodily resonance as providing a convincing account of the processes enabling enactive understanding, while we believe that reflective understanding to some degree relies on all the processes associated with ToM, folk psychology and narrative competence. The significance of these perceptual and interpretive processes in producing empathy, however, is not limited to the epistemic aspects we have focused on so far. They also have a significant impact on the affective attitude we adopt toward others.

Fuchs briefly touches on the other-directed, affective aspects of empathy in distinguishing empathic engagements from other, more objectifying types of interaction (Fuchs, 2013, p 660–661) but he never develops these ideas further. In the following section, we suggest that empathic engagements based on interbodily resonance are in fact characterized by a basic pro-attitude toward the other. We develop this suggestion with inspiration from the ancient Stoics and their theory of *oikeiōsis* or attachment. Based on a theory of perception and intentional action that is closely similar to Fuchs’ concepts of bodily resonance and affective intentionality, these ancient Greek philosophers developed an analysis of interpersonal relations and prosocial motivation that, so we suggest, can usefully supplement Fuchs’ concept of primary empathy and help us understand the affective aspects of this emotional phenomenon.

## Affective Intentionality: Bodily Resonance and Stoic Attachment

The Stoic school established itself as an influential intellectual tradition under the leadership of Zeno, Cleanthes, and Chrysippus in the generations just after Plato and Aristotle and it came to dominate many of the philosophical debates throughout the Hellenistic world well into the second century CE. As part of their overarching project of figuring out how human beings can come to live in perfect harmony with themselves and the world around them, these highly original thinkers developed a sophisticated analysis of how humans orient themselves in the world, their so-called theory of *oikeiōsis*. The structuring idea of this theory is an ability to recognize different things as belonging or proper to oneself (gr. *oikeion*). This, according to the Stoics, produces a sense of attachment (*oikeiōsis*) that structures our perception of the world in much the same way as we perceive our environment in terms of “affective affordances,” i.e., the affective qualities it possesses for us, on Fuchs’ account of affective intentionality.

Based on rather crude, but for their time groundbreaking physical and medical theories, the Stoics describe the perceptual processes underlying this intentionality in highly visceral terms as a consequence of our material soul rubbing against the body, with which it is coextensive and thoroughly blended:

For the soul extends outward with an expansion and strikes all the parts of the body, since it is also mixed with all of them, and when it strikes them it is struck back in turn. For the body too offers resistance, just like the soul: and the affection ends up being simultaneously characterized by pressure and counterpressure [Hierocles, 2009, p. 13 (IV.44–49)].

The affections (*pathē*, a term that also means feelings or emotions) produced through this interaction of body and soul are also described as a reciprocal co-affection between the two—*sympatheia* in ancient Greek [Hierocles, 2009, p .11 (IV.1–22)]. It produces not only an awareness of the various parts of our body but also an instinctual grasp (*antilēpsis*) of their functions and needs [Hierocles, 2009, p. 3–9 (I.1-III.19); and Seneca, 2007, p. 85–89 (121.5–6 and 22–24); with Long, 1996; Brittain, 2002, p. 266–269; Klein, 2016, 172–176]. This, in turn, explains how we perceive external objects. When we encounter something, it affects our body and the soul registers this affection making us aware both of the object and how it affects us [Hierocles, 2009, p. 16 (VI.1–9)]. Depending on whether this affection is perceived to fit with our immediate constitution and needs, it will appear either proper (*oikeion*), foreign (*allotrion*), or neither to us [Hierocles, 2009, p. 9–11 (III.20–54); and Seneca, 2007, 88–89 (121.17–21); with Brittain, 2002, p. 269–271].

The continuous awareness or co-perception (*synaisthesis*) of ourselves also results in a feeling of attachment (*oikeiōsis*) and a basic disposition to show concern (*tērein*) for ourselves—we recognize the various aspects of our constitution as proper to us and therefore start caring for them—and this in turn, is said to produce impulses to pursue things that are *oikeion* and avoid their opposites [Laertius, 1925, p. 193 (VII.85); Cicero, 2001, p. 69–70 (III.16–18); Seneca, 2007, p. 85–89; Hierocles, 2009, 17–21 (VI.28–VII.50); with Klein, 2016, p. 165–178]. An impulse (*hormē*), on the Stoic account, is thus the psychic event causing an animal’s body to move (Inwood, 1985, p. 42–101; Brennan, 2003; Graver, 2007, p. 24–28). For instance, when I am hungry and see a banana, I recognize the banana as suitable nourishment, i.e., as *oikeion* to me, and eating the banana therefore strikes me as appropriate (*kathēkon*) resulting in an impulse to eat the banana.

On the Stoic view, there are thus four psychological mechanisms underlying every action:

1. the perception of something as *oikeion*, *allotrion* or neither through bodily affections/feelings (corresponding to the embodied appraisal of affective affordances on Fuchs’ account of affective intentionality)
2. the natural tendency to feel attachment toward things we recognize as *oikeion*
3. the disposition to show concern (*tērein*) for ourselves and, derivatively, the things we feel attached to
4. the impulse to move (corresponding to Fuchs’ e-motions).^4^

Despite the dualistic coloring of their terminology^5^ the Stoic concept of a *sympatheia* between body and soul therefore bears a close resemblance to Fuchs’ concept of bodily resonance: Both make bodily feelings the medium of affective intentionality and both see these affections as entailing an awareness both of the object affecting you and of yourself being affected, accompanied by a tendency to act. The Stoics, however, add the crucial elements of self-attachment and -concern.

Fuchs’ speaks more neutrally of the self-reference involved in affective intentionality (Fuchs and Koch, 2014; cf. Slaby and Stephan, 2008). This would seem to involve some kind of basic pro-attitude toward oneself—a recognition of myself as “me” and a wish to preserve this self, similar to the self-perception, -attachment, and -concern posited by the Stoics—but this remains somewhat unclear: “They (sc. Emotions) always imply a particular relation to the feeling subject in its very core: through emotions, I experience *how it is for me* to be in this or that situation. To be afraid of an approaching lion (world-reference) means at the same time being afraid for oneself (self-reference). Each emotion, thus, implies the two poles of feeling *something* and feeling *oneself* as inextricably bound together.” (Fuchs and Koch, 2014, p. 3, their emphasis). “Feeling oneself” here seems to include both a perception of oneself (“I experience *how it is for me*”) and a concern for oneself (“being afraid for oneself”). This ambiguity between perception/understanding and concern pervades Fuchs’ account of bodily resonance and to some extent it is inherent in the very words “feeling” and “affection.” The Stoics, on the other hand, effectively dissolve this ambiguity by analyzing the co-perception of the self into the three closely linked, but distinct elements of perception, attachment, and concern: Through my bodily feelings I perceive myself being affected, I recognize this self as something I feel attached to, and therefore I care about what happens to me. The Stoic theory of attachment can therefore helpfully supplement Fuchs’ phenomenological analysis of “incorporation” by providing a terminology for the relational aspects of this way of “having a world” (Merleau-Ponty, 1962, p. 169) and the affective value it supposes us to ascribe to our own body and its surroundings.

As Fuchs stresses incorporation is “a pervasive characteristic of the lived body, which always transcends itself and partly merges with the environment.” (Fuchs and De Jaegher, 2009, p. 472). This includes the unidirectional integration of an object into the sensorimotor schema of the lived body which allows us to interact and coordinate with it as e.g., the blind person incorporates a cane; but it also covers the “intercorporeality” or “mutual incorporation” made possible through the reciprocal interbodily resonance between two living subjects (Fuchs and De Jaegher, 2009, cf. Merleau-Ponty, 1962). The latter type of incorporation, as Fuchs points out, enables us to perceive and respond immediately to the affective states of others, but whereas he goes into considerable detail about the kind of perception he takes to be characteristic of such direct empathy, his account of the affective responses appropriate to empathy remains underdeveloped. The richer Stoic concept of attachment, however, offers the conceptual means for spelling out and clearly delineating the scope of these aspects of empathy.

Not unlike Fuchs, the Stoics held that the *sympatheia* characterizing the interaction of body and soul extends beyond your own organism. In principle, it extends to the entire universe which for them is a compound of body (or matter, *hyle*) and soul (or fiery air, *pneuma*) just like the human organism (Brouwer, 2015). On this basis they seem to have posited some limited possibilities of sharing affective states with other living beings and the world at large (see Brouwer, 2015 for a more detailed discussion), but in contrast to Fuchs, they never seem to have explored the possible epistemic implications of this doctrine^6^. Instead, their analysis of social interaction, like that of self-perception, is carried out in terms of attachment and concern.

While our most fundamental feelings of attachment pertain to ourselves and our constitution, this attitude extends well beyond the borders of our own body to include anything that we recognize as *oikeion* to us. Occasionally this attitude was analyzed into the basic well-disposed (*eunoētikē*) attachment toward ourselves which determines the selective (*hairetikē*) attachment we feel toward external things and the affectionate (*sterktikē*) attachment we feel toward other people (Hierocles, 2009, p. 25 [IX.1-10]; cf. Anonymus, 1995, VII–VIII).

The fact that the Stoics used the same term to describe such different relations suggests that they saw a basic similarity in the character of those relations. The surviving Stoic texts, however, do not offer any univocal explanation of what it is that makes something qualify as *oikeion*—apart from the formal answer that everything that is part of or somehow agrees with our constitution is *oikeion* to us (cf. Klein, 2016)—but the etymology of the term provides some fascinating hints about how the Stoics envisioned the relations it describes. *Oikeion* thus derives from *oikos* (“house” or “household”) and most directly it describes the relation one has toward the people and things one was brought up with. By extension, it came to apply to aspects of one’s own organism. Its Stoic meaning is therefore perhaps best captured by the English “proper” or “akin” (see further Pembroke, 1971). By contrast, the word *idios* (“personal, private, peculiar to you”) more directly describes something that belongs to you as opposed to others^7^. The choice of the word *oikeion* to describe the way we perceive our own organism may thus be taken to reflect a view of the subject as deeply embedded in a social context and community rather than a more or less isolated individual^8^.

Similar to the lived body in phenomenological theories of perception (see e.g., Merleau-Ponty, 1962; Gallagher, 2005, chap. 1), the “self” (in the Stoic sense of the mind-cum-body also referred to as our “constitution”) is the pivotal point of our intentionality. The attachment and concern we feel toward this self furthermore explain the affective value we attribute to the things we perceive and our motivations to care for, pursue or avoid them. As for Fuchs, this perceptual process is pre-reflective and non-verbal. Our perceptions can be verbalized and reflected upon, but they happen through the medium of bodily feelings or affections^9^.

On this view, the attribution of affective value to something, and thereby your concern for it, depends on the establishment of a relation between that object or person and your “self”—on you seeing them as part of or at least relevant to who you are and what you want. But this self is not a stable or monolithic thing. It is subject to constant change and re-configuration during a lifetime, and with it the things and people we recognize as *oikeion* change. The image of the household, at the root of the concept of attachment (or appropriation, as *oikeiōsis* is also frequently translated), is suggestive. Just like the members of your household, your furniture etc. change, and with them the constitution of your household, so your “self” and the things you recognize as belonging to it changes. The late Stoic Hierocles, (2009, p. 91–93 [apud Stob. 4.84.23]; cf. Cicero, 1991, 21–25 [I.50–60]) thus portrays the social world of every human being as structured in circles of ever more attenuated bonds of attachments, from your own body over your closest kin to the most remote stranger.

On its most convincing interpretation, the Stoic theory of attachment therefore reflects what Algra (2003) has called a model of “extended partiality.” On this view, the attachment toward one’s own organism may—at least at the outset of human development—represent a privileged relation, but in principle it is no different from the attachment we feel toward other things that are within our sphere of interest and concern. Attachment, on the Stoic conception, thus transcends the boundaries of the individual agent bringing a broader range of things within our sphere of concern and making them the co-reference point of our actions. It constitutes an extension of our affective intentionality, not only making the other affectively relevant to us but effectively including her interests in our sphere of concern. This model of extended partiality fascinatingly cuts across the entrenched modern distinction between egoistic and altruistic motivations and perforates the borders between “self” and “other” in ways that are suggestive of many modern, enactive, and phenomenological accounts of intersubjectivity and “intercorporeality”—including Fuchs’. The Stoic model of extended partiality, we suggest, therefore provides a promising way of describing the shift in motivations that accompanies the decentering of our intentionality posited by his account.

To sum up, we have argued that in order to fully grasp the significance of interbodily resonance and mutual incorporation in enabling empathic, social engagements one must take into account both its epistemic and affective aspects. We have suggested that the latter be analyzed along the lines of the Stoic theory of attachment and concern. On this account, empathy can be seen to imply a basic pro-attitude toward the other, a perception of the other that presents her as a proper object for our attachment. This, in turn, produces a concern that can generate the prosocial motivation to help her in particular circumstances or a more general attitude of extended partiality toward her. In the following section, we shall relate this model of empathic motivation to the modern debate about the relation between empathy and altruism.

## Discussion: Toward a Comprehensive Conception of Empathy

Having examined both the epistemic and affective aspects of interbodily resonance in some detail, delineating its plausible effects in enabling social understanding and prosocial attitudes, we may now proceed to develop our attempt at a comprehensive account of empathy.

In the section “Empathy: Cognitive, Emotional, Primary, and Extended,” we examined the embodied basis of social understanding in interbodily resonance but argued for the need to acknowledge both a reflective and an enactive type of social understanding. As maintained by Fuchs, enactive understanding quite plausibly supplies the necessary prerequisites for developing ToM and these two ways of engaging with the intentions and perspectives of others remain tightly integrated and mutually informing. Nonetheless, they are different capacities, accounting for different kinds of understanding and empathic engagement: enactive understanding offers an immediate access to the meaning-content of intentional states, while reflective understanding reasons about the subjective meaning and acts of meaning-endowments underlying such states.

Following Fuchs, we see interbodily resonance as fundamental both to direct empathic engagement and to the acquisition of empathic capacities, but with inspiration from the ancient Stoics we have argued that this type of interaction also creates feelings of attachment and concern. On our account, this pro-attitude toward others characterizes any empathic engagement and distinguishes such engagements from more objectifying, disengaged, or “cold-blooded” instances of social understanding. As we argued in the section “Social Understanding: Enactive and Reflective” above, attachment and the entailed recognition of the other as a proper object of concern furthermore suggests a conceptualization of the prosocial behavior often associated with empathy in terms of extended partiality.

**FIGURE 1:**
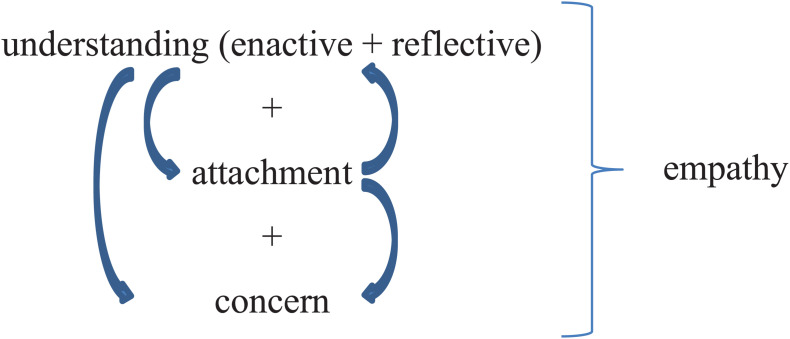
The distinctive elements of empathy. This figure shows empathy as involving social understanding of both the enactive and reflective type, a sense of attachment toward to other and a concern for them that can motivate the empathizer to undertake action in the interest of the target person. Other-concern, we take it, arises on the basis of understanding, attachment, or both (as indicated by the downward arrows in the figure). The arrows between the two latter states indicate that these are most plausibly seen as mutually informing and re-enforcing (attachment can produce an increased propensity to engage in attempts to understand the other, just like an understanding of the other can produce attachment toward them).

Based on these discussions we believe that the relation between the epistemic, affective, and motivational aspects of empathic engagements as in Figure 1.

On this model, empathy is a complex emotional phenomenon that involves three distinct other-related states allowing for varying degrees of intensity and shifting interrelations: an understanding of the other’s affective state, a feeling of attachment toward the other, and a concern for them. It is important to note that the individual processes underlying these states can of course also operate in isolation or appear in other constellations: one can understand the state that someone else is in without feeling neither attachment nor concern toward them (cf. Zahavi’s expert torturer), just like understanding alone can produce concern without any elements of attachment (cf. Bloom’s 2017a,b, concept of “rational compassion”). Similarly, one can feel attachment toward someone else without even trying to understand them, and this can likewise produce concern. What we take to be distinctive of empathy, however, is the co-presence and interplay of these processes and states.

Rather than narrowing the scope of empathy to the imaginative simulation of the other’s affective state, as suggested by de Vignemont and colleagues, or to the immediately perceptual and purely epistemic engagement with others’ states, as suggested by Zahavi, we thus define empathy in virtue of the co-presence of its constitutive elements. Empathy, on our account, therefore differs from mindreading more broadly by implying an attachment and concern for the other. On this account, the mindreading of Zahavi’s expert torturer does not count as empathy; just like her good twin, the rationally compassionate helper who acts out of a fully detached appreciation of someone else’s needs and an impartial, prosocial motivation, does not empathize. Also, we take empathy to be specifically concerned with the affective states of others (or emotions in Fuchs’ broad sense) and only incidentally with non-valenced beliefs and perceptual states.

On the other hand, empathy differs from purely affective types of experiential sharing, like emotional contagion or a collective reaction to some event, by entailing an understanding of the other’s affective state^10^. This epistemic element also distinguishes empathy from more general feelings of pity or sympathy, but if sympathy is conceived broadly as just any benevolent emotional response to others’ affective states, empathy can of course be considered a type of sympathy^11^. Likewise, empathy differs from the mere feeling of concern, that is an element of it, and the motivation to help, that is a frequent consequence of it. On this account, empathy can therefore both be seen as a distinctively affective type of mindreading and a distinctively epistemic type of sympathy.

The distinctive feature of our account is thus the integration of understanding, attachment, and concern as constitutive parts of empathy. This conception of empathy is comprehensive in the sense that it encompasses the epistemic, affective and motivational aspects of empathy. It thereby addresses both of Batson’s questions regarding empathy and insists that a proper answer to either requires us to consider the other as well. This is done on the grounds that social understanding, attachment and concern appear to correlate in ways that elude us when studied in isolation.

Our working definition tries to convey this complexity, in terms that stay neutral with regard to the exact nature of the processes and mechanisms involved, as *a benevolent engagement with the affective states of others which provides us with a grasp of their state and produces an affective response within our bodies*. As mentioned in the section “Empathy: Cognitive, Emotional, Primary, and Extended” above, recent philosophical and empirical debates on empathy have generally focused on questions regarding the scope and importance of different psychological processes in enabling social understanding. Proponents of rivaling accounts, however, tend to agree that a full account of social understanding is likely to involve some combination of all the processes under consideration (Stueber, 2006; O’Shea, 2012; Fuchs, 2017; Gallese and Sinigaglia, 2018; Fernandez and Zahavi, 2020). By merely stipulating that empathy “provides a grasp of the other’s state” our definition allows for different construals of how these processes figure and interact in empathy. In order to clarify the scope of the different proposals, however, we have suggested to distinguish clearly between the attainment of enactive and reflective understanding.

With regard to the former type of understanding, we have pointed to Fuchs’ concept of interbodily resonance and affective intentionality as a convincing way to capture the embodied and enactive character of these processes; but there are also serious attempts to explain this in simulationalist terms (Gallese, 2003; Gallese and Sinigaglia, 2018) just like theory-theory approaches may incorporate many of the objections leveled against it by proponents of phenomenological and 4E approaches (O’Shea, 2012). Our hope is that the account offered here may help advance this debate as it relates to empathy by providing a pluralistic, conceptual framework for studying the epistemic and affective aspects of the relevant processes in combination thereby allowing for a more holistic assessment of their significance in enabling empathic engagements.

We thus take the processes producing empathic understanding and attachment to be mutually informing. As Cialdini et al. (1997) have pointed out an empathic concern deriving from perspective taking, on the one hand, and feelings of oneness, on the other, are likely to arise in the same contexts and they appear sensitive to many of the same factors (relationship closeness, severity of need etc.). This indicates that empathic understanding and attachment are closely correlated and we have pointed to the processes involved in interbodily resonance as important factors in producing both. It is likewise plausible to assume that empathic understanding can create or deepen feelings of attachment, just like feelings of attachment are likely to make you more prone to engage in an attempt to understand the other person (cf. Batson and Shaw, 1991; Cialdini et al., 1997).

Attachment has a long history as a term for describing the intimate bonds between a child and its closest caregivers (see e.g., the seminal paper by Bowlby, 1958) but it has not been used systematically to describe wider social bonds (cf. Batson and Shaw, 1991, p. 113). We have pointed to the Stoic theory of attachment as providing a concise conceptual framework for such an analysis of our social relations. On this theory, our social bonds rely on a proneness to see other people as *oikeion* to us and feel attachment and concern toward them, parallel to the way we perceive our own body and certain aspects of our environment as “belonging” to us. In extension of Fuchs’ adoption of Gibson’s (1979) theory of affordances, one might speak of “empathic affordances” and work toward an understanding of the factors informing this social aspect of our affective intentionality (factors such as kinship, similarity in cultural background, racial prejudice etc. but, perhaps even more importantly, purely bodily factors such as skin-to-skin-contact (Ciaunica, 2017), olfactory intake etc.).

Both the phenomenological concept of incorporation and the Stoic concept of attachment furthermore suggest that our perception of others is in some respects parallel to the way we perceive and relate to our own body. These proprioceptive processes have received increasing attention within contemporary psychology and neuroscience under the umbrella term “Body Ownership” (BO) covering studies of how we attach to/detach from our own limbs and bodies. The quantitative and qualitative studies of BO have highlighted how the sensation of something being my “own” body is dependent on visual and tactile intake and is therefore highly sensitive to manipulation of this intake—even to the degree that such manipulation can create the temporary, illusory adoption of a foreign limb or even an entire body as one’s own (Botvinick and Cohen, 1998; Petkova et al., 2011; Maselli and Slater, 2013; Guterstam et al., 2015).

An important future avenue for empirical studies of empathy, suggested by our account and to some extent already being pursued, is to transfer the insights gained in BO-scholarship about the role of multisensory stimulation in producing feelings of ownership/attachment toward our own body to the intersubjective sphere, on the assumption that they play similar, causal roles in determining how we relate to the bodies of others (cf. Bertrand et al., 2018). Indeed, there is already tentative evidence that adopting another limb (or avatar body) that has a different skin tone also reduces implicit racial biases (Farmer et al., 2012, 2014; Maister et al., 2013; Peck et al., 2013; Farmer and Maister, 2017). The authors of these studies argue that looking through another body and feeling body ownership to it creates a kind of kinship which in turn lowers out-grouping tendencies. These studies provide examples of how even an illusory and temporary perception of someone else as *oikeion*—literally perceiving a foreign limb or body as belonging to you—impacts our attitude toward them, thereby raising important questions about the role of (perceived) similarity or relatedness in creating feelings of attachment, concern, and ultimately empathy, and the importance of pre-reflective, bodily factors in shaping such (a)kinship-perception.

Another important aspect of the Stoic account is the model it provides for describing the prosocial motivation associated with empathy. As brought out by the considerations above, the prosocial motivation that derives from feelings of attachment is inherently partial. It rests on the inclusion of others into your in-group and only thereby into your sphere of concern: the two of us, we Europeans, we humans etc. By contrast, some theorists have assumed that empathy can inspire genuinely altruistic action, i.e., an ultimately selfless motivation to promote the wellbeing of the other induced through perspective taking (Batson and Shaw, 1991). As Cialdini et al. (1997) have convincingly shown, however, feelings of oneness appear to have a far more decisive impact on our decisions to help, suggesting that the potential effects of empathic understanding on prosocial motivation are mediated by such feelings.

This is conveyed by our insistence that empathy necessarily involves a feeling of attachment toward the other and we have described the resulting type of motivation as an extended partiality, i.e., the inclusion of the other into our sphere of concern based on a perception of the other as *oikeion* to us. Herein lies the strengths and limitations of empathy as a motivational force: it brings the affective states and needs of its target into sharp focus, thereby inevitably forcing those of others into the background (cf. Bloom, 2017a,b). Rather than mobilizing us “against empathy,” however, this remarkable capacity of human beings to extend their partiality in our view leaves plenty of room for optimism about the moral value of empathy.

## Conclusion

After a brief review of recent debates about empathy this paper set out to examine and expand upon the enactivist account offered by Thomas Fuchs. Through a critical discussion of his conception of bodily resonance as providing the basis for an immediate understanding of other’s intentional states, we have argued for the need to acknowledge the equal importance of reflective, social understanding in everyday, human interaction and allow for different processes to provide the predominant foundations of these types of understanding.

As a distinctively affective, other-directed intentionality, however, empathy does not merely consist in understanding the affective states of others. It also involves an affective response toward the other and their situation (as the empathizer perceives this). This response, we have suggested, can be analyzed along the lines of the Stoic theory of *oikeiōsis* as a feeling of attachment and concern toward the other that arises on the basis of interbodily resonance along with other, more reflective processes that makes the other appear *oikeion* or (a)kin to us. This inclusion of the other in one’s sphere of concern produces, in turn, a prosocial motivation that can best be described as an extended partiality.

Based on these philosophical reflections we have proposed an account of empathy as a complex emotional phenomenon comprising epistemic, affective, and motivational elements and we have briefly related the Stoic concepts we introduced in order to describe its affective and motivational elements to existing concepts within current debates about Body Ownership and altruistic motivation. Our approach has been shamelessly eclectic—integrating concepts from ancient Stoicism, modern phenomenology, psychology, and neuroscience—but the result, we submit, is an account of empathy that acknowledges the complexities of this other-directed and inherently partial way of engaging with others and can thereby help increase conceptual clarity across the interdisciplinary field of empathy studies.

## Data Availability Statement

All datasets generated for this study are included in the article/supplementary material.

## Author Contributions

The ideas for this article along with a first draft were developed in close collaboration between the authors. The final version was written by TS with comments and suggestions from KB. Both authors contributed to the article and approved the submitted version.

## Conflict of Interest

The authors declare that the research was conducted in the absence of any commercial or financial relationships that could be construed as a potential conflict of interest.

## Footnotes

1 We shall use the term “mindreading” in the neutral sense of any capacity to register the intentional states of others, not as referring to a purely mentalistic understanding.

2 On de Vignemont’s account, condition (v) is introduced in order to account for the modulation of empathy by contextual and personal factors but it remains somewhat unclear what this condition involves apart from the fact that de Vignemont takes empathy to be incompatible with “an egotist stand” and likely to be blocked by an indifferent or negative attitude toward the target person (de Vignemont and Jacob, 2012, p. 307). We elaborate the benevolence we take empathy to imply below, sections “Social Understanding: Enactive and Reflective” and “Affective Intentionality: Bodily Resonance and Stoic Attachment.”

3 Note that this question presupposes a more fundamental question about how we can know *that* others are thinking, sometimes referred to as “the problem of other minds.” We shall not address this issue here.

4 A note of clarification: The analogy that we suggest is between Fuchs’ bodily feelings and the so-called “hormetic,” i.e., impulse-producing, impressions (*hormētikai phantasiai*), not the subspecies of misguided reactions that the Stoics provocatively called emotions (Graver, 2007, p. 15–34). The Stoics thus analyzed the reaction to multisensory stimuli into two distinct psychic events: impressions, defined as an “alteration” or “affection (*pathos*) that happens in the soul and shows in itself the thing that produced it” [Long and Sedley, 1987, p. 237 (39B, Aëtius 4.12.2); with Brittain, 2002, p. 259–261]; and impulses (*hormē*), defined as a “movement” in the soul, which follows upon these affections when they are productive of impulses (Inwood, 1985, p. 42–66; Brennan, 2003). When this reaction is appropriate, it results in a “proper act” (*kathēkon*), when it is inappropriate, in an emotion (*pathos* again). In adults, these reactions presuppose a reflective act of “assent” to the hormetic impression (whereas in children and animals it follows directly on the impressions), but besides these (at least potentially) reflective reactions the Stoics also recognized a set of pre-reflective and therefore uncontrollable reactions called pre-emotions (*propatheiai*), i.e., bodily reactions which are independent of our assent (Graver, 2007, p. 85–108). Parallel to Fuchs, we suggest, the Stoics thus see bodily affections/feelings as constituting an embodied appraisal of the environment in terms of *oikeion, allotrion* or neutral, which entails both an affection (the impression) and expression (the pre-emotion) caused by the external stimuli, while the impulses following upon this appraisal correspond to Fuchs’ concept of a movement tendency or “e-motion.”

5 In overt rejection of Platonic dualism, the Stoics in fact stress the holistic unity of body, soul and intellectual mind (Gill, 2006). Cf. Fuchs’ rejection of “the Cartesian framework” of traditional cognitive science (e.g., Fuchs and De Jaegher, 2009, p. 468; Fuchs, 2017, p. 27–30).

6 The Stoics were e.g., (in)famous for claiming that in the ideal case one would be able to feel the effect of another person stretching out her finger at the other end of the world and share the joy of this movement if it was carried out well [Plutarch, 1976, p. 730–731 (Comm. not. 1068F)]. This, however, would be a case of sharing (in Scheler’s sense of feeling the same due to the same event) and therefore involves no social understanding.

7 *Idios* is the etymological root of the, often derogatory, term *idiōtēs*, which signifies the private citizen as opposed to the citizen actively taking part in the duties of the political community. Interestingly, we find [Plato (1997), 1,089 (462b)] coining the word *idiōsis* (“privatization” from *idios*, parallel to *oikeiōsis* from *oikeios*), contrasting it with *koinōnia*, “community.”

8 The Stoics were of course picking up on already established general and philosophical usages of the term *oikeion* (although they did to all appearances use the term far more systematically than any of their predecessors and thereby probably helped extend its meanings). To some degree the communal mindset we ascribe to them here was thus part of their cultural background. For a good discussion of how this mind set is reflected in Stoic social philosophy, see Algra (2003).

9 Whereas Fuchs conceives of bodily feelings as non-representational (see e.g., Froese and Fuchs, 2012, p. 207, 213), however, this does not seem to be the case with the Stoics (Sorabji, 1993, p. 20–28; Brittain, 2002).

10 Note, however, that emotional contagion and sharing, on our account, can lead to empathy *if* they bring about an awareness that the other is experiencing a similar affective state *and* this awareness is accompanied by a feeling of attachment and concern toward them.

11 Sympathy carries shifting meanings, many of which overlap with the concept of empathy, but often it simply appears as an umbrella term for benevolent emotional responses to others (for a good overview, see Stueber, 2006, p. 28–31).

## References

[B1] Algra K. (2003). The mechanism of social appropriation and its role in hellenistic ethics. *Oxf. Stud. Anc. Philos.* 25 265–296.

[B2] Anonymus (1995). “Commentarium in platonis theaetetum,” in *Corpus Dei Papiri Filosofici Greci e Latini*, Vol. 3 eds Sedley D. Bastianini G. (Leuven: Leuven University Press) 221–562.

[B3] Batson C. D. (2009). “These things called empathy: eight related but distinct phenomena,” in *The Social Neuroscience of Empathy*, eds Decety J. Ickes W. (Cambridge, MA: The MIT Press).

[B4] Batson C. D. Shaw L. L. (1991). Evidence for altruism: toward a pluralism of prosocial motives. *Psychol. Inq.* 2 107–122. 10.1207/s15327965pli0202_1

[B5] Bertrand P. Guegan J. Robieux L. McCall C. A. Zenasni F. (2018). Learning empathy through virtual reality: multiple strategies for training empathy-related abilities using body ownership illusions in embodied virtual reality. *Front. Robot. AI* 5:26 10.3389/frobt.2018.00026 PMC7805971 33500913

[B6] Bloom P. (2017a). *Against Empathy: The Case for Rational Compassion.* New York, NY: Random House.

[B7] Bloom P. (2017b). Empathy and its discontents. *Trends Cogn. Sci.* 21 24–31. 10.1016/j.tics.2016.11.004 27916513

[B8] Botvinick M. Cohen J. (1998). Rubber hands ‘Feel’ touch that eyes see. *Nature* 391 756–756. 10.1038/35784 9486643

[B9] Bowlby J. (1958). The nature of the child’s tie to his mother. *Int. J. Psycho Anal.* 39 350–373. 13610508

[B10] Brennan T. (2003). “Stoic moral psychology,” in *The Cambridge Companion to the Stoics*, ed. Inwood B. (Cambridge: Cambridge University Press).

[B11] Brittain C. (2002). Non-rational perception in the stoics and augustine. *Oxf. Stud. Anc. Philos.* 22 253–308.

[B12] Brouwer R. (2015). “Stoic sympathy,” in *Sympathy: A History*, ed. Schliesser E. (Oxford: Oxford University Press), 15–35. 10.1093/acprof:oso/9780199928873.003.0002

[B13] Carruthers P. Smith P. K. (1996). *Theories of Theories of Mind.* Cambridge: Cambridge University Press.

[B14] Cialdini R. B. Brown S. L. Lewis B. P. Luce C. Neuberg S. L. (1997). Reinterpreting the empathy–altruism relationship: when one into one equals oneness. *J. Pers. Soc. Psychol.* 73:481 10.1037/0022-3514.73.3.481 9294898

[B15] Ciaunica A. (2017). The ‘Meeting of Bodies’ – empathy and basic forms of shared experiences. *Topoi* 38 185–195. 10.1007/s11245-017-9500-x

[B16] Cicero M. T. (1991). in *On Duties*, ed. Griffin trans. M. T. Atkins E. M. (Cambridge: Cambridge University Press). 10.1007/s11245-017-9500-x

[B17] Cicero M. T. (2001). in *On Moral Ends*, ed. Annas trans. J. Woolf R. (Cambridge: Cambridge University Press). 10.1007/s11245-017-9500-x

[B18] De Jaegher H. Pieper B. Clénin D. Fuchs T. (2017). Grasping intersubjectivity: an invitation to embody social interaction research. *Phenomenol. Cogn. Sci.* 16 491–523. 10.1007/s11097-016-9469-8

[B19] de Vignemont F. Jacob P. (2012). What is it like to feel another’s pain? *Philos. Sci.* 79 295–316.

[B20] de Vignemont F. Singer T. (2006). The empathic brain: how, when and why? *Trends Cogn. Sci.* 10 435–441. 10.1016/j.tics.2006.08.008 16949331

[B21] Farmer H. Maister L. (2017). Putting ourselves in another’s skin: using the plasticity of Self-perception to enhance empathy and decrease prejudice. *Soc. Justice Res.* 30 323–354. 10.1007/s11211-017-0294-1

[B22] Farmer H. Maister L. Tsakiris M. (2014). Change my body, change my mind: the effects of illusory ownership of an outgroup hand on implicit attitudes toward that outgroup. *Front. Psychol.* 4:1016. 10.3389/fpsyg.2013.01016 24454301 PMC3888940

[B23] Farmer H. Tajadura-Jiménez A. Tsakiris M. (2012). Beyond the colour of my skin: how skin colour affects the sense of body-ownership. *Conscious. Cogn.* 21 1242–1256. 10.1016/j.concog.2012.04.011 22658684 PMC3772504

[B24] Fernandez A. V. Zahavi D. (2020). Basic empathy: developing the concept of empathy from the ground up. *Int. J. Nurs. Stud.* 110:103695. 10.1016/j.ijnurstu.2020.103695 32736251

[B25] Froese T. Fuchs T. (2012). The extended body: a case study in the neurophenomenology of social interaction. *Phenomenol. Cogn. Sci.* 11 205–235. 10.1007/s11097-012-9254-2

[B26] Fuchs T. (2013). The phenomenology and development of social perspectives. *Phenomenol. Cogn. Sci.* 12 655–683. 10.1007/s11097-012-9267-x

[B27] Fuchs T. (2016). “Intercorporeality and interaffectivity,” in *Intercorporeality: Emerging Socialities in Interaction*, eds Meyer C. Streeck J. Jordan J. S. (Oxford: Oxford University Press), 194–209.

[B28] Fuchs T. (2017). “Levels of empathy–primary, extended, and reiterated empathy,” in *Empathy*, eds Lux V. Weigel S. (Berlin: Springer), 27–47. 10.1057/978-1-137-51299-4_2

[B29] Fuchs T. De Jaegher H. (2009). Enactive intersubjectivity: participatory sense-making and mutual incorporation. *Phenomenol. Cogn. Sci.* 8 465–486. 10.1007/s11097-009-9136-4

[B30] Fuchs T. Koch S. C. (2014). Embodied affectivity: on moving and being moved. *Front. Psychol.* 5:508. 10.3389/fpsyg.2014.00508 24936191 PMC4047516

[B31] Gallagher S. (2004). Understanding interpersonal problems in autism: interaction theory as an alternative to theory of mind. *Philos. Psychiatry Psychol.* 11 199–217. 10.1353/ppp.2004.0063

[B32] Gallagher S. (2005). *How the Body Shapes the Mind.* Oxford: Oxford University Press, 10.1093/0199271941.001.0001

[B33] Gallagher S. (2012a). Empathy, simulation, and narrative. *Sci. Context* 25 355–381. 10.1017/s0269889712000117

[B34] Gallagher S. (2012b). “Neurons, neonates and narrative,” in *Moving Ourselves, Moving Others*, eds Foolen Ad Lüdtke U. M. Racine T. P. Zlatev J. (Amsterdam: Benjamins), 167–196.

[B35] Gallese V. (2003). The roots of empathy: the shared manifold hypothesis and the neural basis of intersubjectivity. *Psychopathology* 36 171–180. 10.1159/000072786 14504450

[B36] Gallese V. Sinigaglia C. (2018). “Embodied resonance,” in *The Oxford Handbook of 4E Cognition*, eds Newen A. Bruin L. De Gallagher S. (Oxford: Oxford University Press), 10.1093/oxfordhb/9780198735410.013.22

[B37] Gangopadhyay N. (2014). Introduction: embodiment and empathy, current debates in social cognition. *Topoi* 33 117–127. 10.1007/s11245-013-9199-2

[B38] Gibson J. J. (1979). *The Ecological Approach to Visual Perception: Classic Edition.* East Sussex: Psychology Press.

[B39] Gill C. (2006). *The Structured Self in Hellenistic and Roman Thought.* Oxford: Oxford University Press.

[B40] Goldman A. (2006). *Simulating Minds The Philosophy, Psychology, and Neuroscience of Mindreading.* New York, NY: Oxford University Press, 10.1093/0195138929.001.0001

[B41] Goldman A. de Vignemont F. (2009). Is social cognition embodied? *Trends Cogn. Sci.* 13 154–159. 19269881 10.1016/j.tics.2009.01.007

[B42] Gopnik A. Wellman H. M. (1992). Why the child’s theory of mind really is a theory. *Mind Lang.* 7 145–171. 10.1111/j.1468-0017.1992.tb00202.x

[B43] Graver M. R. (2007). *Stoicism* & *Emotion.* Chicago, IL: University of Chicago Press.

[B44] Guterstam A. Björnsdotter M. Gentile G. Ehrsson H. H. (2015). Posterior cingulate cortex integrates the senses of self-location and body ownership. *Curr. Biol.* 25 1416–1425. 10.1016/j.cub.2015.03.059 25936550

[B45] Hierocles (2009). *Hierocles the Stoic: Elements of Ethics, Fragments and Excerpts.* trans. I. Ramelli and D. Konstan Atlanta, GA: Society of Biblical Lit.

[B46] Hutto D. D. (2004). The limits of spectatorial folk psychology. *Mind Lang.* 19 548–573. 10.1111/j.0268-1064.2004.00272.x

[B47] Inwood B. (1985). *Ethics and Human Action in Early Stoicism.* New York, NY: Oxford University Press.

[B48] Jensen R. T. Moran D. (2012). Introduction: intersubjectivity and empathy. *Phenomenol. Cogn. Sci.* 11 125–133. 10.1007/s11097-012-9258-y

[B49] Klein J. (2016). The stoic argument from Oikeisis. *Oxf. Stud. Anc. Philos.* 50 143–200. 10.1093/acprof:oso/9780198778226.003.0005

[B50] Laertius D. (1925). *Lives of Eminent Philosophers, Volume II* Loeb Classical Library 185. trans. Hicks R. D. Cambridge, MA: Harvard University Press.

[B51] León F. Zahavi D. (2016). “Phenomenology of experiential sharing: the contribution of schutz and walther,” in *The Phenomenological Approach to Social Reality*, eds Salice A. Schmid H. (cham: Springer), 219–234. 10.1007/978-3-319-27692-2_10

[B52] Long A. A. (1996). “Hierocles on oikeiōsis and self-perception,” in *Stoic Studies*, ed. Long (Berkeley, CA: Cambridge University Press), 250–263.

[B53] Long A. A. Sedley D. N. (1987). *The Hellenistic Philosophers.* Cambridge: Cambridge University Press, 1.

[B54] Lyons-Ruth K. Bruschweiler-Stern N. Harrison A. M. Morgan A. C. Nahum J. P. Sander L. (1998). Implicit relational knowing: its role in development and psychoanalytic treatment. *Infant Ment. Health J.* 19 282–289. 10.1002/(sici)1097-0355(199823)19:3<282::aid-imhj3>3.0.co;2-o

[B55] Maister L. Sebanz N. Knoblich G. Tsakiris M. (2013). Experiencing Ownership over a dark-skinned body reduces implicit racial bias. *Cognition* 128 170–178. 10.1016/j.cognition.2013.04.002 23680793 PMC3750641

[B56] Maselli A. Slater M. (2013). The building blocks of the full body ownership illusion. *Front. Hum. Neurosci.* 7:83. 10.3389/fnhum.2013.00083 23519597 PMC3604638

[B57] Merleau-Ponty M. (1962). *Phenomenology of Perception.* Abingdon: Routledge.

[B58] Newen A. De Bruin L. Gallagher S. (2018). *The Oxford Handbook of 4E Cognition.* Oxford: Oxford University Press.

[B59] O’Shea J. R. (2012). The ‘theory theory’of mind and the aims of sellars’ original myth of jones. *Phenomenol. Cogn. Sci.* 11 175–204. 10.1007/s11097-011-9250-y

[B60] Peck T. C. Seinfeld S. Aglioti S. M. Slater M. (2013). Putting yourself in the skin of a black avatar reduces implicit racial bias. *Conscious. Cogn.* 22 779–787. 10.1016/j.concog.2013.04.016 23727712

[B61] Pembroke S. G. (1971). “Oikeiosis,” in *Problems in Stoicism*, ed. Long A. A. (Oxford: Athlone Press), 126.

[B62] Petkova V. I. Björnsdotter M. Gentile G. Jonsson T. Li T. Ehrsson H. H. (2011). From part- to whole-body ownership in the multisensory brain. *Curr. Biol.* 21 1118–1122. 10.1016/j.cub.2011.05.022 21683596

[B63] Plato (1997). *Complete Works*, eds Cooper J. M. Hutchinson D. S. (Indianapolis, IN: Hackett Publishing).

[B64] Plutarch (1976). *Moralia, Volume XIII: Part 2: Stoic Essays* Loeb Classical Library 470, ed. Henderson J. (Cambridge, MA: Harvard University Press). trans. H. Cherniss.

[B65] Premack D. Woodruff G. (1978). Does the chimpanzee have a theory of mind? *Behav. Brain Sci.* 1 515–526. 10.1017/s0140525x00076512

[B66] Scheler M. (1954). *The Nature of Sympathy.* trans. P. Heath Abingdon: Routledge.

[B67] Schmidsberger F. Löffler-Stastka H. (2018). Empathy is proprioceptive: the bodily fundament of empathy–a philosophical contribution to medical education. *BMC Med. Educ.* 18:69. 10.1186/s12909-018-1161-y 29622015 PMC5887217

[B68] Schutz A. (1967). *The Phenomenology of the Social World.* Evanston, IL: Northwestern University Press.

[B69] Seneca L. A. (2007). *Seneca: Selected Philosophical Letters - Translated with Introduction and Commentary.* trans. B. Inwood Oxford: University Press, Incorporated.

[B70] Slaby J. Stephan A. (2008). Affective intentionality and self-consciousness. *Conscious. Cogn. Soc. Cogn. Emot. Self Conscious.* 17 506–513. 10.1016/j.concog.2008.03.007 18417362

[B71] Sorabji R. (1993). *Animal Minds and Human Morals: The Origins of the Western Debate.* Ithaca, NY: Cornell University Press.

[B72] Stueber K. (2006). *Rediscovering Empathy: Agency, Folk Psychology, and the Human Sciences.* Cambridge, MA: Institute of Technology.

[B73] Zahavi D. (2008). Simulation, projection and empathy. *Conscious. Cogn. Soc. Cogn. Emot. Self Conscious.* 17 514–522. 10.1016/j.concog.2008.03.010 18411058

[B74] Zahavi D. (2010). Empathy, embodiment and interpersonal understanding: from lipps to schutz. *Inquiry* 53 285–306. 10.1080/00201741003784663

[B75] Zahavi D. (2011). Empathy and direct social perception: a phenomenological proposal. *Rev. Philos. Psychol.* 2 541 10.1007/s13164-011-0070-3

[B76] Zahavi D. (2014). Empathy and other-directed intentionality. *Topoi* 33 129–142. 10.1007/s11245-013-9197-4

[B77] Zahavi D. Michael J. (2018). “Beyond mirroring: 4E perspectives on empathy,” in *The Oxford Handbook of 4E Cognition*, eds Newen A. De Bruin L. Gallagher S. (Oxford: Oxford University Press), 589–606.

[B78] Zaki J. Ochsner K. N. (2012). The neuroscience of empathy: progress, pitfalls and promise. *Nat. Neurosci.* 15 675–680. 10.1038/nn.3085 22504346

